# Percutaneous Debulking of a Large Right Atrial Tumor

**DOI:** 10.1016/j.jscai.2022.100412

**Published:** 2022-08-07

**Authors:** Bassim El-Sabawi, Ashley Mohadjer, Dean Holiday, Pete P. Fong

**Affiliations:** a Division of Cardiovascular Medicine, Vanderbilt University Medical Center, Nashville, Tennessee; b Department of Pathology, Vanderbilt University Medical Center, Nashville, Tennessee

**Keywords:** angiosarcoma, AngioVac, intracardiac mass, thrombectomy

A 57-year-old woman presented with altered mentation and hypotension. One month prior, she underwent pulmonary endarterectomy for presumed chronic thromboembolic pulmonary hypertension, where she was found to have a mass in the left pulmonary artery. Pathology revealed intimal angiosarcoma. An intraoperative transesophageal echocardiogram (TEE) showed no intracardiac masses.

A transthoracic echocardiogram and computed tomography of the chest revealed a new 4.7-cm mass in the right atrium (RA) (Figure 1A, B and Supplemental Video 1). She was declined for surgical resection after evaluation by the Heart Team because of poor oncologic prognosis with metastatic disease and increased surgical risk with recent sternotomy and pancytopenia from chemotherapy. She was referred for palliative transcatheter debulking given the rapid tumor growth and potential for hemodynamic collapse from obstruction across the tricuspid valve or massive pulmonary embolism.

**Figure 1:**
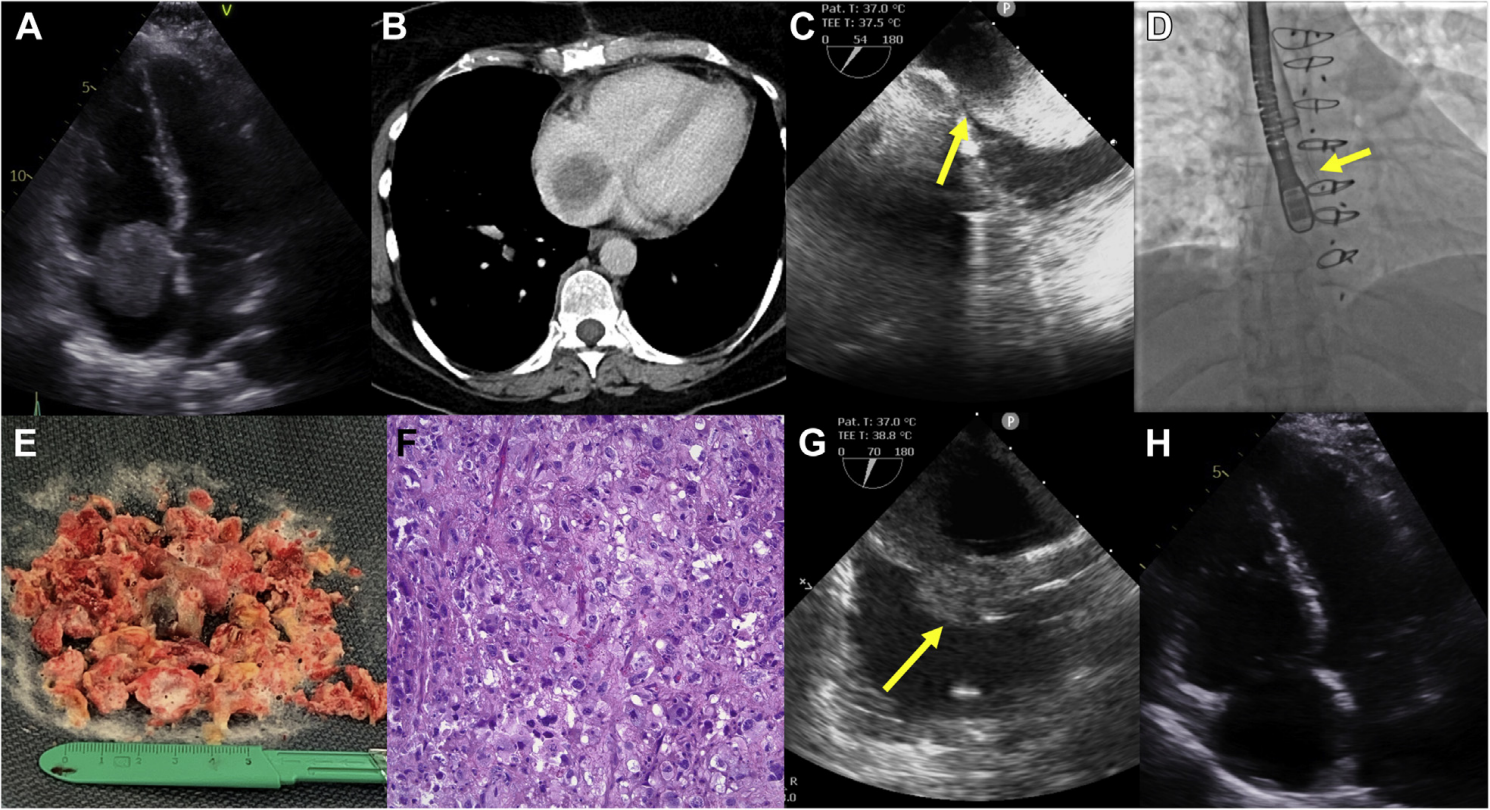
**Transcatheter right atrial tumor debulking using the AngioVac device.** (**A**) Apical 4-chamber view demonstrating a 4.7-cm right atrial mass protruding through the tricuspid valve. (**B**) Cross-sectional computed tomography demonstrating a large filling defect in the right atrium. (**C**) Modified view from the intraprocedural TEE showing the mass attached to the inferior vena cava and right atrial junction (yellow arrow). (**D**) Fluoroscopy showing the AngioVac device (yellow arrow) in the right atrium. (**E**) Gross image of the tissue retrieved. (**F**) High power magnification (200×) of tumor cells demonstrating marked nuclear atypia. (**G**) Modified TEE view postintervention showing the remaining stalk of the tumor (yellow arrow). (**H**) Postprocedure apical 4-chamber view demonstrating that the mass was no longer evident. TEE, transesophageal echocardiogram.

The procedure was performed under general anesthesia with TEE and fluoroscopic guidance. TEE demonstrated a pedunculated mass attached to the inferior vena cava and RA junction (Figure 1C). A 26F DrySeal sheath (Gore Medical) was placed in the right internal jugular vein, and an 18F venous return cannula was placed in the left femoral vein and attached to the extracorporeal bypass circuit. A 22F AngioVac (AngioDynamics) aspiration cannula was connected to the bypass circuit and inserted into the right internal jugular vein via the 26F sheath. The AngioVac funnel tip was deployed and maneuvered to engage the large RA mass. Pump flow was initiated up to 3 L/min. Numerous sweeps were performed using a “rotating push and pull” technique to break up the mass with the suction catheter (Figure 1D and Supplemental Video 2), which was successful in gradual debulking. Intermittent “clamp and release” technique was used on the outflow cannula, creating a transient increase in suction to facilitate the removal of larger portions of the mass. Tissue retrieved from the device filter was grossly consistent with tumor, and histologic examination revealed sheets of tumor cells with background tumor-type necrosis and marked nuclear atypia consistent with the spread of the previously diagnosed metastatic intimal angiosarcoma (Figure 1E, F). An estimated 80% of the mass was successfully removed, with only a portion of the stalk remaining on TEE (Figure 1G). The patient tolerated the procedure well. The mass was no longer evident and right ventricular systolic function was normal on a postprocedure transthoracic echocardiogram (Figure 1H and Supplemental Video 3). She had no in-hospital complications and was discharged with improved hemodynamics.

One month later, the patient returned with similar symptoms. Repeat imaging demonstrated recurrence of the large RA mass despite palliative chemotherapy. She was deemed not to be a candidate for further cancer-directed therapies and died in hospice a few days later.

Percutaneous aspiration thrombectomy is being increasingly used with expanding indications, including intracardiac masses (thrombus, vegetation, or neoplasm), pulmonary embolus, and venous or arterial thrombosis.^1^ Acceptable safety and efficacy have been reported using the AngioVac system for these indications in a recent multicenter registry.^2^ The comparative differences between the various available large bore aspiration thrombectomy devices are shown in Table 1. Percutaneous aspiration of tumor carries additional challenges due to the difficulty of breaking-up tissue for aspiration and the risk of embolization. Prior cases of percutaneous debulking of tumor-thrombus using the AngioVac system have been reported with variable success.^3^, ^4^, ^5^ Our case illustrates the feasibility and technical aspects of this approach for gradual debulking of a very large right atrial tumor without a thrombotic component using this system.

**Table 1:** Comparison of the characteristics of available large bore aspiration thrombectomy devices.

Device	Thrombectomy mechanism	Estimated blood loss	Cannula size	Comments
AngioVac system (AngioDynamics, Inc)	Continuous mechanical suction via the extracorporeal venovenous circuit	<250 mL	Inflow: 18F or 22F catheter Return: 18F catheter	Continuous suction and filtered return of blood may be beneficial in the extirpation of larger (>2 cm) masses while minimizing blood loss.
Indigo system (Penumbra, Inc)	Continuous mechanical suction via the external engine	Approximately 250-750 mL	7F, 8F, or 12F catheter	Improved torqueability and patient comfort with smaller catheters. System limited by continuous blood loss during aspiration.
FlowTriever system (Inari Medical)	Manual aspiration via a suction syringe. Option of mechanical extirpation using nitinol disks	<250 mL	16F, 20F, or 24F catheter	High vacuum pressures can be obtained with this system. Minimal blood loss with the use of the FlowSaver blood return system.

## Supplementary material

Video 1 Apical 4-chamber view demonstrating a large right atrial mass prolapsing into the tricuspid valve.

Video 2 Intraprocedural transesophageal echocardiography showing the AngioVac device suction of the right atrial mass.

Video 3 Postintervention apical 4-chamber view revealing that the right atrial mass was no longer evident.
